# *Oenocarpus bacaba* and *Oenocarpus bataua* Leaflets and Roots: A New Source of Antioxidant Compounds

**DOI:** 10.3390/ijms17071014

**Published:** 2016-06-27

**Authors:** Louis-Jérôme Leba, Christel Brunschwig, Mona Saout, Karine Martial, Didier Bereau, Jean-Charles Robinson

**Affiliations:** Université de Guyane, UMR QUALITROP, Campus Universitaire de Troubiran, P.O. Box 792, 97337 Cayenne Cedex, French Guiana, France; louis-jerome.leba@univ-guyane.fr (L.-J.L.); christel.brunschwig@gmail.com (C.B.); mona.saout@gmail.com (M.S.); karine.martial@univ-guyane.fr (K.M.); didier.bereau@univ-guyane.fr (D.B.)

**Keywords:** *Oenocarpus bacaba*, *Oenocarpus bataua*, roots, leaf, antioxidant activity, LC-MS/MS, C-glycosyl flavones, hydroxycinnamic acids, cellular assay

## Abstract

Native palm trees fruit from the Amazonian rainforest, *Oenocarpus bacaba* and *Oenocarpus bataua*, are very often used in the diet of local communities, but the biological activities of their roots and leaflets remain poorly known. Total phenolic content (TPC) and antioxidant activity of root and leaflet extracts from *Oenocarpus bacaba* and *Oenocarpus bataua* were assessed by using different chemical assays, the oxygèn radical absorbance capacity (ORAC), the 2,2-diphenyl-l-picrylhydrazyl (DPPH) free radical-scavenging capacity and the ferric-reducing ability of plasma (FRAP). Cellular antioxidant activity and cytotoxicity were also measured in Normal Human Dermal Fibroblasts. The polyphenolic composition of *Oenocarpus* extracts was investigated by LC-MS^n^. *Oenocarpus* leaflet extracts were more antioxidant than root extracts, being at least as potent as *Euterpe oleracea* berries known as superfruit. *Oenocarpus* root extracts were characterized by hydroxycinnamic acids (caffeoylquinic and caffeoylshikimic acids), while leaflet extracts contained mainly caffeoylquinic acids and C-glycosyl flavones. These results suggest that leaflets of both *Oenocarpus* species could be valorized as a new non-cytotoxic source of antioxidants from Amazonia, containing hydroxycinnamic acids and flavonoids, in the pharmaceutical, cosmetic or agri-food industry.

## 1. Introduction

**Figure 1:**
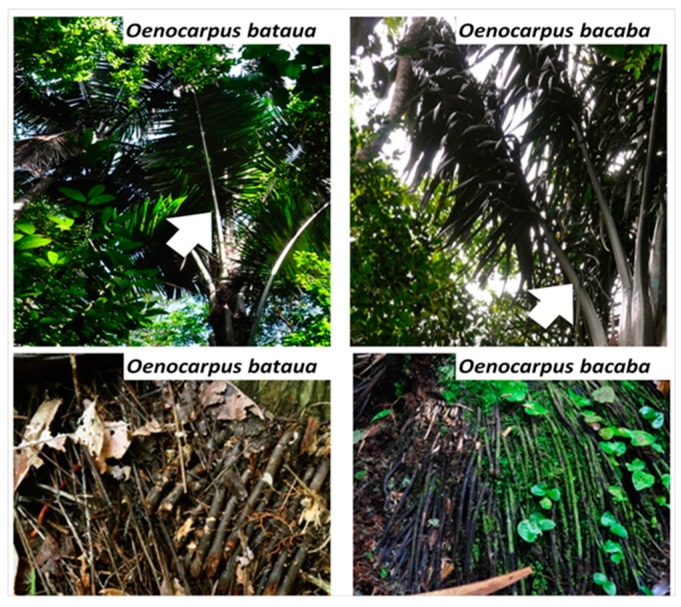
Organs collected from *Oenocarpus bataua* and *Oenocarpus bacaba* in French Guiana. **Upper panel** show the leaves selected for leaflets collection (white arrow); **Lower panel** show roots selected for collection.

When dealing with antioxidant topics, significant attention is currently paid to the characterization of antioxidant activity of fruit and vegetables available in land [1,2,3]. This increasing focus is mainly due to the capacity of fruit and vegetables components to reduce oxidative stress level in cells [4]. Oxidative stress is commonly defined as a disorder in the equilibrium status, between pro-oxidant and antioxidant systems in healthy cells, leading to oxidative damage of cellular components like proteins, lipids, and DNA [5]. The alteration of cellular components by oxidative stress is now strongly associated with pathological dysfunctions within the human organism like cancers [6,7], cardiovascular and immune diseases [8,9,10], neurodegenerative diseases like Alzheimer’s and Parkinson’s [11,12], metabolic syndromes induced by diabetes and obesity [13,14] and cells ageing [15]. The main agents of oxidative stress in human cells are considered to be the byproducts of aerobic metabolism leading to the synthesis of Reactive Nitrogen Species (RNS) and Reactive Oxygen Species (ROS), among them can be found nitric oxide (·NO), peroxynitrite (ONOO^−^), hydrogen peroxide (H_2_O_2_), superoxide ($O_{2}^{-}$) and hydroxyl radicals (·OH) [16,17]. Over the past decade in South America, a great attention has been paid to identify Amazonian fruits and plants with antioxidant potential [18,19,20,21]. From these investigations, Amazonian berries produced by the palm tree *Euterpe oleracea*, well known as acai, have been identified as super fruits due to their high antioxidant activity [22]. Many studies of *E. oleracea* berries have followed since [23,24,25,26] and the commercial success of *E. oleracea* berries across the world has shifted the focus away from other palm berries commonly consumed by Amazonian people. In French Guiana, in addition to the berries of *E. oleracea,* berries of other palm trees like *Oenocarpus bataua* and *Oenocarpus bacaba*, locally called patawa and comou, are frequently used by local communities. While substantial bibliography is available on the biological activities of *E. oleracea* [27,28], only few articles have focused on *O. bacaba* and *O. bataua*. Moreover, these studies focused on the berries [19,20], while the biological activity and the chemical composition of their vegetative organs remain poorly known. Indeed, only Galotta et al. partially investigated the chemical composition and antioxidant activities of *E. precatoria*‘s vegetative organs using the 2,2-diphenyl-l-picrylhydrazyl (DPPH) and β-carotene assays [29,30]. Actually, roots and leaflets of Amazonian palm trees (*E. oleracea* and *E. precatoria*) are cited for the treatment of diabetes, for kidney and liver pains, fever, and used against snake bites [31,32] showing that these vegetative organs might have interesting biological activity and compounds. In this study, we therefore assessed the antioxidant properties of leaflets and roots of *O. bacaba* and *O. bataua* (Figure 1). Three solvents were selected for extraction: water (W), acetone/water 70/30 *v*/*v* (A) and methanol/water 70/30 *v*/*v* (M). Water was selected for environmental reasons (eco-friendly), whereas mixed solvents like acetone/water and methanol/water were selected for their high extraction yield especially in polyphenols [33,34,35]. Total polyphenolic contents were measured by the Folin-Ciocalteu method [36] and the antioxidant activity was assessed using different chemical assays involving various mechanisms of action, substrates and radicals: the 2,2-diphenylpicrylhydrazyl (DPPH) free radical-scavenging capacity [37], the Ferric-Reducing Ability of Plasma (FRAP) [38] and the Oxygen Radical Absorbance Capacity (ORAC) (peroxide radical) [39]. The extracts were also assessed in a cell-based assay, the Cellular Antioxidant Assay (CAA). To better understand and identify the compound involved in the antioxidant activity, extracts were analyzed by LC-MS^n^. Results from these assays are expected to give a better insight into the antioxidant activity and phytochemical composition of roots and leaflets of *O. bacaba* and *O. bataua*.

## 2. Results and Discussion

### 2.1. Total Phenolic Content (TPC)

The acetone/water and methanol/water extracts had average to high TPC (25 to 60 µg GAEq/mg DM) as expected (Table 1). The extracts with the highest TPC were the acetone leaflet extracts of *O. bacaba* and *O. bataua*, as well as the methanol leaflet extracts of *O. bacaba* (Table 1). Overall, leaflet extracts were more concentrated in polyphenols than the corresponding root extracts. Compared to other palm trees, vegetables and plants extracts, their TPC was equivalent to that of *O. bataua*, *E. oleracea* berries [19] and *O. bacaba* berries [20], 3-fold higher than lettuce, one of the richest vegetable in the study of Tiveron et al. [40], and half that of green tea leaves [41], as one of the plants with the highest known antioxidant activity, thought to be linked to its polyphenolic compounds [42,43,44,45].

### 2.2. Chemical Antioxidant Assays

Antioxidant properties of *Oenocarpus* leaflet and root extracts were investigated using different chemical assays, oxygen radical absorbance capacity (ORAC_FL_), the 2,2-diphenyl-1-picrylhydrazyl (DPPH) free radical-scavenging capacity and the ferric-reducing ability of plasma (FRAP) working according to different modes of action. *Oenocarpus* extracts were potent in the free radical scavenging assays (DPPH, ORAC_FL_), and in reducing Fe(III) into Fe(II) (FRAP) (Table 2). In the DPPH assay, acetone leaflet extracts of *O. bataua* and *O. bacaba* and water leaflet extracts of *O. bacaba* were the most active with values ranging from 461 to 545 µmol Teq/g DM (Table 2 and Table S1), which were 2-fold higher than that of *E. oleracea* and *O. bataua* berries, with already high antioxidant activities [19] (Table S2). The best extract had a DPPH value 0.5-fold lower than that of green tea measured by Leba et al. [41] (1185 µmol Teq /g DM), (Table 2 and Table S2). Interestingly enough, the two less potent extracts (*O. bataua* water root and leaflet extracts) (Table 2) had a DPPH activity similar to the most potent extracts in the study of Tiveron et al. [40], i.e., lettuce and artichoke (77 ± 1 and 70 ± 5 µmol Teq/g DM, respectively).

In the FRAP assay, the three best extracts (acetone leaflet extracts of *O. bataua* and *O. bacaba* as well as methanol leaflet extract of *O. bacaba*) had values ranging from 729 to 1026 µmol Fe(II)Eq/g DM (Table 2 and Table S1), which were 1.5- to 2-fold higher than that of palm berries [19] or antioxidant foodstuff like lettuce [40] but two fold lower than that of acetone and methanol extracts of green tea (Table 2 and Table S2).

The ORAC_FL_ assay, considered as one of the most relevant antioxidant chemical assays, was performed with the palm extracts. Once again, the most potent extracts in ORAC_FL_ assay were acetone leaflet extracts of *O. bataua* and *O. bacaba* and methanol leaflet extract of *O. bacaba* with 1200–1565 µmol Teq/g DM (Table 2 and Table S1). Their ORAC values were at least 2-fold higher than that of *E. oleracea* and *O. bataua* berries [19]. Interestingly, the best ORAC value (1565 µmol Teq/g DM for of *O. bacaba*-A) was only 1.5-fold lower than that of green tea with 2366 µmol Teq/g DM (Table 2 and Table S2). This study points out that Amazonian palm tree roots and leaflets have chemical antioxidant activities as high as palm berries. These extracts were then assessed in a cell-based antioxidant assay.

### 2.3. Cellular Antioxidant Activity (CAA) and Cytotoxicity

Regarding the promising results in terms of antioxidant activity in the chemical assays, *Oenocarpus* extracts were then assessed in a cell-based antioxidant assay. None of the root or leaflet extracts of *O. bataua* and *O. bacaba* were cytotoxic to Normal Human Dermal Fibroblasts (NHDF) at a concentration of 100 µg/mL (Table 3) and were therefore tested at this concentration in the cellular antioxidant activity assay with NHDF cells. EC_50_ of *Oenocarpus* extracts ranged from 10 to 74 µg/mL, most of them being more active than fruits or vegetables known as good antioxidants in a similar cell-based assay like kiwis, blueberries, boiled carrots and strawberries with EC_50_ from 50 to 200 µg/mL [46]. This is the first time that leaflets and root extracts of *Oenocarpus* species have been reported to be active in a cell-based assay with promising activity.

When converted into µmoles Quercetin Eq/g DM and µmoles Quercetin Eq/100 g FW, the most potent extracts were the acetone leaflet extracts of *O. bacaba* and *O. bataua* with 50 and 101 µmoles Quercetin Eq/g DM and with 2172 and 5029 µmoles Quercetin Eq/100 g FW, respectively. Interestingly, there was a very good correlation between the ORAC_FL_ activity and the CAA activity (Pearson’s *r* correlation coefficients of 0.897) (Figure S1).

This previously reported correlation between ORAC and CAA activity [46], suggests that *Oenocarpus* extracts may act in the CAA mainly through a mechanism of peroxyl radical scavenging, which is the mechanism of the ORAC assay. If the active compounds present in the extracts were able to cross the cell membrane, these results could have interesting implications in the dermatological field, as human dermal fibroblasts are connective tissues, which maintain the structural framework of the tissues and play a critical role in wound healing. Further in vitro evaluation of *Oenocarpus* extracts as radical scavengers or as anti-inflammatory agents is needed, since oxidative stress can also activate various inflammatory pathways [47].

### 2.4. Correlations Between Total Phenolic Contents and Antioxidant Activities

High Pearson’s *r* correlation coefficients (*p* < 0.05) were found between TPC and DPPH assay (0.946) (Figure S2a), between TPC and FRAP assay (0.986) (Figure S2b) between TPC and ORAC assay (0.989) (Figure S2c), and between TPC and CAA assay (0.953) (Figure S2d).

This emphasizes the link between the polyphenolic content and the antioxidant activity, which is not typically found in similar studies dealing with vegetables [40] or herbal teas [42]. Good dispersion of our data due to wide ranges of TPC and antioxidant values in relation to the different organs and extraction solvent selected, might explain the high Pearson’s *r* correlation coefficients found across all antioxidant assays (>0.9). These results indicate that *Oenocarpus* extracts were acting according to different modes of action in the different antioxidant assays and that this antioxidant activity was linked to their polyphenolic composition.

### 2.5. Identification of Compounds in Oenocarpus Root and Leaflet Extracts by LC-MS^n^

**Figure 2:**
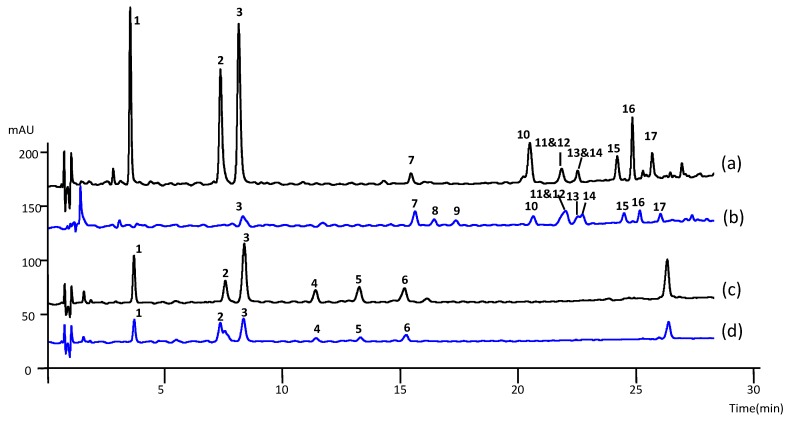
Representative chromatograms of (**a**) leaflet extracts of *Oenocarpus bataua*; (**b**) leaflet extracts of *Oenocarpus bacaba*; (**c**) root extracts of *Oenocarpus bataua*; (**d**) root extracts of *Oenocarpus bacaba* at λ = 320 nm; Kinetex PFP column, (100 × 4.6) mm, 2.6 µm. (**1**) 3-CQA, (**2**) 4-CQA, (**3**) 5 CQA, (**4**) 4-CSA, (**5**) 5-CSA, (**6**) CSA, (**7**) 6,8-di-C-hexosyl apigenin, (**8**,**9**) 6,8-di-C-hexosyl apigenin sulfate, (**10**,**14**,**15**), 6-C-hexosyl-8-C-pentosyl apigenin isomers, (**12**,**16**) 6-C-pentosyl-8-C-hexosyl apigenin isomer, (**11**) 8-C-glucosyl luteolin, (**13**) 6-C-glucosyl luteolin, (**17**) 6-C-glucosyl apigenin.

To better understand the antioxidant activity of *O. bataua* and *O. bacaba* roots and leaflets, their polyphenolic composition was investigated by UV and LC-MS^n^. The UV chromatograms recorded at 320 nm showed 14 main peaks for leaflet extracts and 6 for root extracts (Figure 2), whose structures were proposed using LC-MS^n^ data.

#### 2.5.1. Characterization of Caffeoylquinic Derivatives (*M*_r_ = 354) in Root and Leaflet Extracts

In the root and leaflet extracts, three peaks (**1**, **2** and **3**) at *t*_R_ 4.2, 7.2 and 9.1 min gave [M − H]^−^ ions at *m*/*z* 353 and [M + Na]^+^ at *m*/*z* 377 and showed UV spectra (at λ = 298 and 320 nm) corresponding to caffeoylquinic acids (CQA, *M*_r_ = 354). According to their fragment ions at *m*/*z* 191, 179 and 173 in negative mode [48], compound **1** was assigned as 3-CQA, compound **2** as 4-CQA, compound **3** as 5-CQA (Table 4).

#### 2.5.2. Characterization of Caffeoylshikimic Derivatives (*M*_r_ = 336) in Root Extracts

In the *Oenocarpus* root extracts, three peaks (**4**, **5** and **6**) at t_R_ 12.2, 14.2 and 16.2 min yielded [M − H]^−^ ions at *m*/*z* 335, [M + Na]^+^ at *m*/*z* 359, and showed UV spectra (λ = 298 and 320 nm) corresponding to caffeoylshikimic acids (CSA, *M*_r_ = 336).

The structure of CSA was tentatively assigned according to their diagnostic ions at *m*/*z* 317, 291, 179, 161 and 135 in negative mode [49,50] (Table 4). Compound **4** was identified as 4-CSA, compound **5** as 5-CSA, while the exact structure of compound **6** could not be determined due to inconclusive data.

#### 2.5.3. Characterization of Apigenin Derivatives (*M*_r_ = 432, *M*_r_ = 594, *M*_r_ = 674, *M*_r_ = 564) in Leaflet Extracts

In the *Oenocarpus* leaflet extracts, eleven compounds (**7**–**17**) showed a λ_max_ at 340 nm, typical of flavone derivatives.

Compound **17** (*M*_r_ = 432) showed [M − H]^−^ at *m*/*z* 431 and [M + H]^+^ at *m*/*z* 433 and losses of 90, 120 and 150 Da, characteristics of hexose residues. Compound **17** was confirmed to be 6-C-glucosyl apigenin due to fragment ions at *m*/*z* 311 (aglycone + 42) and *m*/*z* 341 (aglycone + 72) in negative mode [51], and using a synthetic standard.

Nine compounds, compound **7** (*M*_r_ = 594), *compounds **8**, **9*** (*M*_r_ = 674), compounds **10**, **12**, **14, 15** and **16** (*M*_r_ = 564) showed characteristic MS^2^ fragmentation of di-C-glycosyl flavones in negative mode. Fragment ions at *m*/*z* 353 (aglycone + 83) and 383 (aglycone + 113) were indicative of apigenin derivatives [51].

Compound **7** (*M*_r_ = 594) yielded [M − H]^−^ at *m*/*z* 593, and losses of 90 and 120 Da characteristic of hexose derivatives were in concordance with a 6,8-di-C-hexosyl apigenin. 

Compounds **8** and **9** (*M*_r_ = 674) yielded [M − H]^−^ at *m*/*z* 673 and [M + K]^+^ at 713. The MS^2^ spectra showed a first loss of 80 Da, characteristic of sulfates, followed by typical losses of hexose residues (90, 120 and 150 Da), were indicative of 6,8-di-C-hexosyl apigenin sulfates [52]. 

Six peaks (**10**, **12**, **14**, **15**, **16**) (*M*_r_ = 564) at *t*_R_ 21.2, 22.3, 23.2, 24.9 and 25.6 min yielded [M − H]^−^ ions at *m*/*z* 563 in the negative mode. Fragment ions corresponding to neutral losses of 60, 90, 120 and 150 Da, were characteristic of pentose and hexose residues and suggested 6,8-di-C-pentosyl-hexosyl apigenin derivatives. Compounds **12** and **16** were identified as 6-C-pentosyl-8-C-hexosyl apigenin isomers, due to the higher intensity of the fragment [M − H-60]^−^ ion (*m*/*z* 503) resulting from the fragmentation of the pentose moiety on position 6, which fragments preferentially over sugars on position 8 [51]. Others compounds (**10**, **14** and **15**) were assigned as 6-C-hexosyl-8-C-pentosyl apigenin isomers.

#### 2.5.4. Characterization of Luteolin Derivatives (*M*_r_ = 448)

Compounds **11** and **13** (*M*_r_ = 432) gave [M − H]^−^ at *m*/*z* 447, [M + H]^+^ at *m*/*z* 449 and showed UV spectra (λ = 270 and 340 nm) characteristics of flavones. Fragment ions at *m*/*z* 327 (aglycone + 42) and *m*/*z* 357 (aglycone + 72) in negative mode, and typical losses of 90 and 120 Da clearly indicated C-glycosyl luteolin derivatives [51]. Retention order and MS^2^ data clearly indicated compound **11** as 8-C-glucosyl luteolin and compound **13** as 6-C-glucosyl luteolin. This was confirmed using synthetic standards.

### 2.6. Chemical Composition of Root and Leaflet Extracts

**Figure 3:**
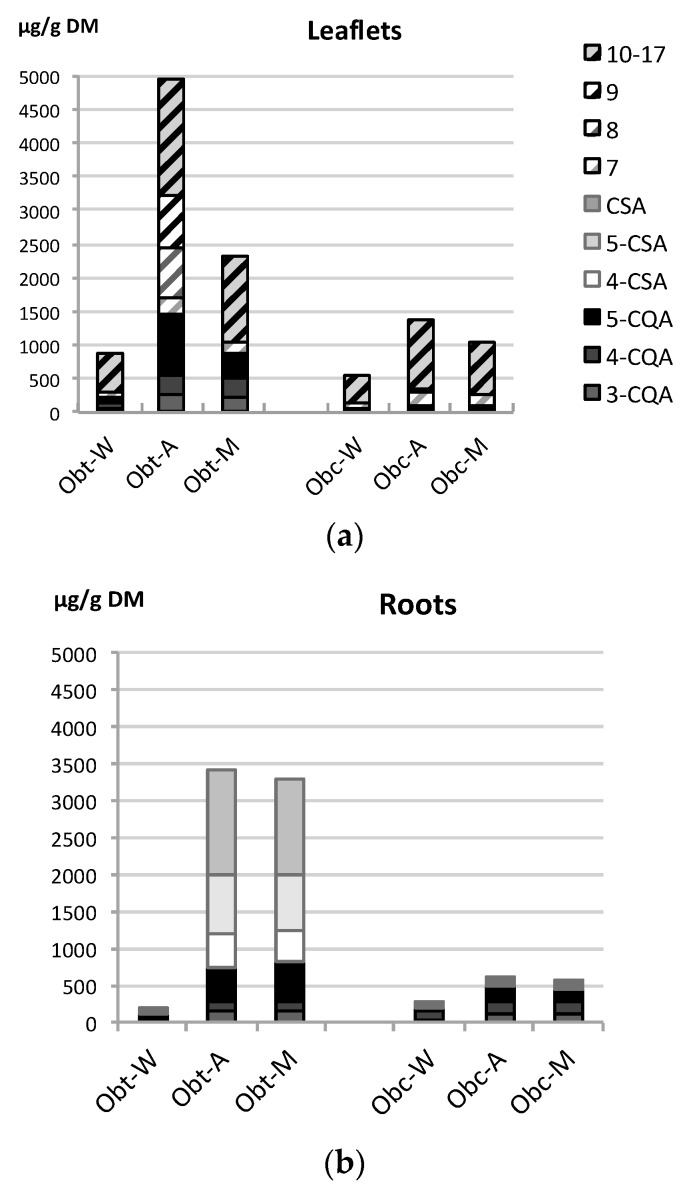
Chemical composition of (**a**) leaflet extracts and (**b**) root extracts of *Oenocarpus bataua* and *Oenocarpus bataua*. Obt: *Oenocarpus bataua*; Obc: *Oenocarpus bacaba*; W: water; A: acetone/water: 70/30; M: methanol/water: 70/30; CQA: caffeoylquinic acid; CSA: caffeoylshikimic acid (for identification of the compounds 7 to 17, see Table 4); *n* = 3 biological replicates.

C-glycosyl flavones and caffeoylquinic acids were the main constituents of *O. bataua* and *O. bacaba* leaflet extracts (Figure 3a). The overall flavone content (compounds **7**–**17**) was similar between the two *Oenocarpus* species (from 500 to 1400 µg/g DM), except when *O. bataua* leaflets were extracted with acetone where a 2- to 3-fold increase in flavone content (3500 µg/g DM) was measured compared to *O. bacaba*. In fact, C-glycosyl flavones are ubiquitous flavonoids in plants, especially within the monocotyledons including Arecaceae, the palm tree family [53]. The flavone profiles (type of flavone aglycone, sugar moiety, substitution pattern) provide valuable taxonomic markers to differentiate species. The flavonoid patterns of *O. bataua* and *O. bacaba* leaflet extracts were similar, characterized mainly by the presence of C-glycosyl apigenin accounting for 60% to 80% of all flavones. This flavonoid pattern is less frequent within the Arecaceae family, where C-glycosyl luteolin derivatives are more common [54]. Thus, C-glycosyl apigenin derivatives could be used as phytochemical markers of *O. bataua* and *O. bacaba*.

The CQA pattern of leaflet extracts was more pertinent to differentiate the two *Oenocarpus* species. The CQA content was 5–10-fold higher in *O. bataua* than in *O. bacaba* leaflet extracts (200–1400 µg/g DM against 40–100 µg/g DM), no matter the extraction solvent (Figure 3a). Overall, acetone and methanol leaflet extracts gave higher yields in flavones and CQA for both *Oenocarpus* species.

The root extracts of *O. bacaba* and *O. bataua* were differentiated according to their hydroxycinnamic acid profiles, i.e., CQA and CSA. *O. bacaba* roots contained mainly CQA (which accounted for 80% of all HCA, irrespective of the solvent used), while *O. bataua* roots showed a mixed CQA-CSA profile, with a CQA content accounting between 20%–70% of all HCA (Figure 3b). In all extracts, 5-CQA was the major caffeoylquinic acid. Regarding these data, the caffeoylshikimic acids could be used as chemical markers to differentiate *O. bataua* roots from *O. bacaba* roots. The presence of hydroxycinnamic acids at high concentration in vulnerable organs like the roots may be linked to their important physiological role of defence against pathogens and disease resistance [55,56]. The differences in HCA composition of the two *Oenocarpus* species may also greatly impact their capacity to interact with their biotic environment and influence their resistance or symbiose spectrum.

### 2.7. Relationship between Chemical Composition and Antioxidant Activity

**Figure 4:**
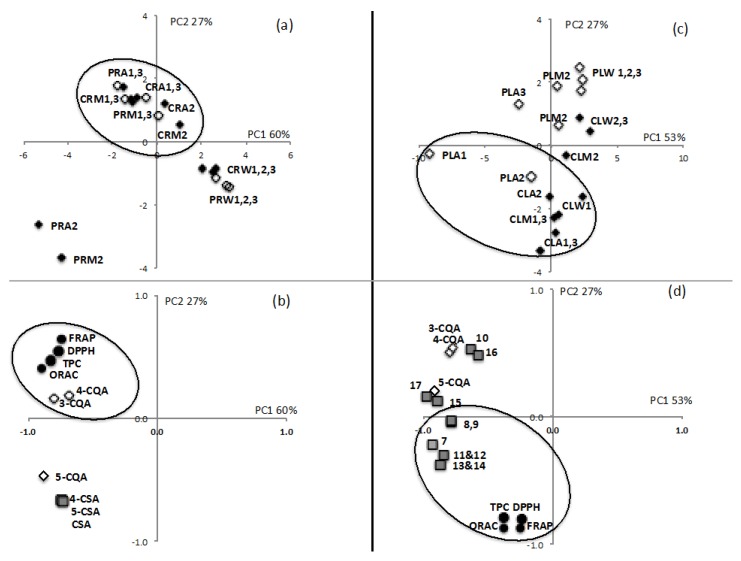
Principal component analysis plots for antioxidant activity and chemical composition of (**a**,**b**) root extracts and (**c**,**d**) leaflet extracts of *Oenocarpus bacaba* and *Oenocarpus bataua*. C: Comou (*Oenocarpus bacaba*), P: patawa (*Oenocarpus bataua*); R: roots; L: leaflets; W: water; A: acetone/water: 70/30; M: methanol/water: 70/30.

As good correlations were found between Total Polyphenolic Contents and antioxidant activity in the different assays (Figure 3), a Principal Component Analysis was performed to identify the compounds mainly involved in the antioxidant activity of *Oenocarpus* root and leaflet extracts. In the root extracts, high values in the DPPH, FRAP, ORAC and TPC assays (negative values of PC1) were correlated with high contents of 3-CQA and 4-CQA rather than with CSA content (Figure 4a,b). Despite high CSA content in *O. bataua* roots, their antioxidant activity was equivalent to that of *O. bacaba* roots. Therefore, the CQA content was found to be the main driver for high antioxidant activity in the *Oenocarpus* roots*.*

It is less clear regarding the PCA results of the *Oenocarpus* leaflets, to identify which compounds were the main drivers for antioxidant activity. Overall, it seems that some of the flavones were correlated to high antioxidant activity rather than the CQA (Figure 4c,d). Indeed, luteolin and apigenin derivatives are known to have high antioxidant activity due to the presence of a hydroxyl substituent [57].

## 3. Experimental Section

### 3.1. Ethics Statement

The study was carried out on private land, whose owner agreed with the collection of vegetative organs of *O. bataua* and *O. bacaba*. These palms are used locally by French Guiana’s population. Moreover, *O. bacaba* and *O. bataua* are not protected or considered endangered plants species.

### 3.2. Plant Material

Leaflets and adventitious roots from *O. bacaba* and *O. bataua* were collected in January 2013 in the city of Macouria, 2 km away from Cayenne, French Guiana. Three biological replicates were collected for each *Oenocarpus* species. Roots and leaflets were free of physical damages, injuries from insects and fungal infections. All sample*s* were freeze-dried and ground using a Thermomix TM 31 (Vorwerk, France). The dried matter was then stored at −20 °C to limit degradation until analysis.

### 3.3. Extraction

Three solvents were selected for extraction of palm roots and leaflets: water (W), acetone/water (A) (70/30, *v*/*v*) and methanol/water (M) (70/30, *v*/*v*), freeze-dried powders (2.5 g) were extracted four times in 50 mL of solvent under sonication (130 kHz, 10 min) at room temperature. Then, each extract was centrifuged (5000× *g*, 10 min, 4 °C) and supernatants were combined after filtration. Organic solvents were evaporated in a rotary evaporator using a bath at 40 °C. Aqueous extracts were lyophilized. Finally, dried extracts were weighed to determine their yield and dissolved in the extraction solvent at a concentration of 40 mg/mL.

### 3.4. Chemicals

Analytical grade quality methanol, acetone and ethanol were used (Carlo Erba, Fontenay-aux-Roses, France). Fluorescein disodium salt, 2,4,6-tris(2-pyridyl)-*s*-triazine (TPTZ), 2′,7′-dichlorofluorescin diacetate (DCFH-DA) and glacial acetic acid were obtained from Fluka Sigma–Aldrich (Steinheim, Germany). 2,2-Diphenyl-1-picrylhydrazyl (DPPH), 6-hydroxy-2,5,7,8-tetramethylchromane-2-carboxylic acid (Trolox), 2,2′-azobis(2-methylpropionamidine) dihydrochloride (AAPH) and quercetin came from Acros Organics (Geel, Belgium). K_2_HPO_4_, KH_2_PO_4_ and FeCl_3_ came from Fisher Scientific (Villebon sur Yvette, France). Gallic acid and iron(II) heptahydrate sulfate were purchased from Alfa Aesar (Ward Hill, MA, USA). Folin–Ciocalteu reagent (Reuil, France), dimethyl sulfoxide (DMSO), Na_2_CO_3_, ethylenediaminetetraacetic acid disodium salt dihydrate (EDTA-Na_2_) and hydrochloric acid came from Carlo Erba reagents (Reuil, France). Hydrogen peroxide and sodium acetate trihydrate came from Chimix (Chassieu, France).

### 3.5. Phytochemical Analysis

#### 3.5.1. Total Phenolic Content (TPC)

The total phenolic content of *Oenocarpus* extracts was determined according to the Folin-Ciocalteu procedure with some modifications of the protocol developed by Arnous et al. [36] or V. L. Singleton and Joseph A. Rossi [58]. Briefly, 100 μL of appropriate dilutions of palm tree extracts and 450 μL of Folin-Ciocalteu reagent were mixed into 2300 μL of distilled water. 150 μL of a 20% sodium carbonate (Na_2_CO_3_) solution was added and incubated for 2 h at room temperature in the dark. The UV absorbance was recorded at 750 nm using a Cary^®^ 50 UV-Vis spectrophotometer (Varian, INC, Les Ulis, France). Gallic acid was used as a reference standard. The TPC was expressed in µg gallic acid equivalent per milligram of dried matter (µg GAEq/mg DM).

#### 3.5.2. Analysis by Liquid Chromatography Mass Spectometry (LC-MS/MS)

Liquid Chromatography Mass Spectometry (LC-MS/MS) analysis was performed on an ion trap (500-MS Varian, Varian, INC, Les Ulis, France) equipped with an electrospray ionization source and coupled to a Pro Star Dynamax LC system (Varian, INC, Les Ulis, France) and a Pro Star 335 PDA (Agilent). Extracts were filtered using 0.2 µm PTFE filters. 20 µL of samples were injected onto a Kinetex PFP column, (100 × 4.6) mm, 2.6 µm (Agilent) using a gradient of 1% formic acid (A) and acetonitrile (B) at 25 °C. The gradient was as follows: 5%–10% B in 10 min, 10%–20% in 10 min, 20% held for 10 min, 20%–100% in 5 min, 100% held for 5 min, and returned to initial conditions for 9 min to re-balance the column. The flow rate was 1 mL/min. The total run time per sample was 60 min.

Analyses were first performed using Full scan mode (*m*/*z* 120–750), both in negative mode and positive mode to identify the molecular ions and then in TurboDDS™ mode (Data Dependent Scanning) to acquire MS^2^ spectra. Helium was used as the damping and collision gas at 0.8 mL/min. Under negative ionization mode, the operation parameters were as follows: nebulizer gas: air, nebulizer gas pressure 50 psi, drying gas pressure 25 psi, drying gas temperature 350 °C, needle voltage −5000 V, spray shield voltage −600 V, spray chamber 50 °C, capillary voltage 100 V. Under positive ionization mode, the operation parameters were as follows: nebulizer gas nitrogen, nebulizer gas pressure 50 psi, drying gas pressure 25 psi, drying gas temperature 350 °C, needle voltage 5000 V, spray shield voltage 600 V, spray chamber 50 °C, capillary voltage 70 V. Enhanced mass spectrum was used as a survey scan to trigger information dependent acquisition of MS/MS spectra. The criteria for the data-dependent acquisition (IDA) were set for the most two intense peaks, which exhibited more than 5000 counts. The identification of compounds was performed according to their MS^2^ fragmentation, using standards when available.

Quantification was carried out with data acquired at 320 nm from the PDA, using gallic acid as internal standard (280 nm). 5-CQA and 6-C-glucosyl apigenin (isovitexin) were used as calibration standards to quantify hydroxycinnamic acids (caffeoylquinic acids CQA and caffeoylshikimic acids CSA) and flavones, respectively.

### 3.6. Chemical Antioxidant Assays

Chemical antioxidant properties were assessed using DPPH, FRAP and ORAC assays as described in our previous paper, Leba et al. [41]. These assays are based on different antioxidant mode of actions like electron transfer, hydrogen transfer or a mixed transfer implying both electron and hydrogen transfer.

#### 3.6.1. 2,2-Diphenyl-1-picrylhydrazyl free radical-scavenging capacityAssay (DPPH)

DPPH radical scavenging activity of the extracts were measured using the method adapted from Brand-Williams et al. [37]. Five dilutions of the extracts were prepared. An aliquot of 100 μL of each dilution was added in triplicates to 3900 μL of DPPH solution (0.1 mM) and vortexed. A methanolic solution of DPPH was used as a control and Trolox as a reference standard. After 2 h of incubation in the dark at room temperature, the absorbance of controls and samples was measured at 520 nm using a Cary^®^ 50 UV-Vis spectrophotometer (Varian, INC). The DPPH radical scavenging activity was expressed in μmoles Trolox equivalent/g of dried matter (TEq μmol/g DM).

#### 3.6.2. Ferric-Reducing Ability of Plasma Assay (FRAP)

The FRAP assay was performed using the original method of Benzie and Strain [38], with some modifications. A FRAP solution was prepared by adding 25 mL of acetate buffer (pH 3.6 at 300 mM), 2.5 mL of acid TPTZ solution (10 mM in HCl at 40 mM) and 2.5 mL of FeCl_3_ (20 mM in water) freshly prepared. 3000 µL of this FRAP solution was mixed with 300 μL of distilled water and 100 μL of reference standard (Fe(II) as Fe_2_SO_4_) or test sample at three different concentrations in triplicates. The absorbance was measured at 595 nm after 30 min at 37 °C using a Cary^®^ 50 UV-Vis spectrophotometer (Varian, INC). Results were expressed in mmol Fe(II) equivalent/g of dried matter (mmol Fe(II) Eq/g DM).

#### 3.6.3. Oxygen Radical Absorbance Capacity Assay (ORAC)

The ORAC procedure was done according to the method of Ou et al. [59] with some modifications. The assay was carried out on a Cary Eclipse Fluorescence Spectrophotometer (Varian, France) in phosphate buffer pH 7.4 (75 mM) at 37 °C. Fluorescein was used as fluorescent probe, AAPH as a peroxyl radical generator and Trolox as a standard at 20–120 µM. 200 µL of blank, standard or sample (diluted in 75 mM phosphate buffer) and 2 mL of fluorescein 30 nM were mixed and pre-incubated at 37 °C for 15 min. Then, 200 µL of 153 mM of AAPH were added. Fluorescence was measured every minute for 60 min at an emission wavelength of 520 ± 5 nm and an excitation wavelength of 485 ± 5 nm using a Cary Eclipse Fluorescence Spectrophotometer (Varian, INC). All samples were analyzed at three different dilutions and run in duplicates with a relative standard deviation <10%. The quantification of the antioxidant activity was based on the calculation of the area under the curve deduced from that of the blank. Antioxidant activity was expressed as µmol of trolox equivalents/g of dried matter (µmol TEq/g DM).

### 3.7. In Cellulo Assay

#### 3.7.1. Cell Culture

Normal Human Dermal Fibroblasts cells (NHDF) were purchased from PromoCell (Heidelberg, Germany). NHDF cells were cultured in growth medium (RPMI GlutaMAX™ (89%), supplemented with 5% FBS (Fetal Bovine Serum), and 1% of antibiotics. Cells were maintained at 37 °C and cultured in 5% CO_2_ as recommended by the manufacturer. The fibroblasts were used for cytotoxicity assay and cellular antioxidant assay as described below.

#### 3.7.2. Cytotoxicity Assay

First, cytotoxicity assays in NHDF cells were performed to determine the maximum concentration of palm tree extracts to use for the CAA assay so as to ensure the viability of the NHDF cells. The cytotoxicity was measured using the 3-(4,5-dimethylthiazol-2-yl)-2,5-diphenyltetrazolium bromide (MTT) method adapted from Ferreira Silva et al. [26]. Briefly, NHDF cells incubated for 24 h at 37 °C, in presence or absence of palm extracts, were washed twice with 500 µL of a phosphate buffer solution (PBS). Then a MTT solution at 0.5 mg/mL was added to the culture medium. NHDF cells were placed in an incubator at 37 °C during 3 h. The MTT solution was removed from the plate using 1 mL of DMSO and the plate was kept in the dark at room temperature during 30 min. Absorbance was recorded at 595 nm using a plate-reader (Dynex Magellan Biosciences, Chantilly, Virginia, VA, USA). The cytotoxicity of the extracts was evaluated at five different concentrations (100, 200, 300, 400 and 500 µg/mL). Extract concentrations, for which a decrease in absorbance by more than 20% was observed, were considered as cytotoxic to the NHDF cells.

#### 3.7.3. Cellular Antioxidant Assay

As all palm extracts were not toxic or weakly toxic to NHDF cells at a concentration of 100 µg/mL, this concentration was therefore chosen as the maximum concentration for the CAA assay using NHDF cells. The CAA assay was done according to Wolf and Liu [60] with some modifications. Briefly, Normal Human Dermal Fibroblasts cells were seeded at a density of 11 × 10^4^ cells by well on a 96-well microplate in 100 μL of growth medium by well. Twenty-four hours after seeding, the growth medium was removed and the wells were washed with PBS. Triplicate wells were treated for 1 h with 100 μL of palm tree extract at 10, 25, 50 and 100 µg/mL plus 50 μM DCFH-DA dissolved in treatment medium and kept in the dark. Quercetin was used as a reference standard. Each plate included triplicate control and blank wells: control wells contained cells treated with 50 µM of DCFH-DA; blank wells contained cells treated only with growth medium. Then the wells were drained from the treatment medium and 100 μL of AAPH at 250 µM was added in each well (excepted in blank wells). Immediately after the AAPH addition, the plate was placed into a plate-reader Dynex Magellan Biosciences (Denkendorf, Germany) at 37 °C and the fluorescence was recorded during 30 min. Emission was measured at a wavelength of 538 nm with an excitation wavelength of 485 nm. After blank subtraction from the fluorescence readings, the area under the curve of fluorescence versus time was integrated to calculate the CAA value at each concentration of palm extracts tested as follows: CAA unit: 100 − (∫SA/∫CA) × 100 where ∫SA is the integrated area under the sample fluorescence versus time curve and ∫CA is the integrated area from the control curve. The median effective dose EC_50_, was determined for palm extracts from the median effect plot of log (*ƒ*_a_/*ƒ*_u_) versus log (concentration), where ƒ_a_ is the fraction affected and *ƒ*_u_ is the fraction unaffected by the treatment. The EC_50_ values were expressed in μg/mL. The EC_50_ of palm extracts were then converted in µmol Quercetin Eq/g DM and μmol Quercetin Eq/100g of FW using the equation log (ƒ_a_/ƒ_u_) = *f* (log(concentration)) for comparison with literature data.

### 3.8. Statistical Analysis

Results were presented as the mean of three biological replicates and standard error (SE) or standard error of the mean (SEM). Comparisons between palm extracts in the CAA, ORAC, FRAP and DPPH assays were performed using an ANOVA followed by multiple comparisons using Fisher’s least significant difference test. Differences were considered to be significant when *p* < 0.05.

## 4. Conclusions

Leaflet extracts of *O. bataua* and *O. bacaba,* and to a lesser extent root extracts*,* were highly potent in the chemical antioxidant assays (DPPH, FRAP, ORAC) and this translated well to the cellular antioxidant assay with Normal Human Dermal Fibroblasts. These results suggest that extracts were able to work through a mechanism of free radical scavenging in a cellular environment. *Oenocarpus* leaflets were characterized by a complex flavonoid pattern, composed mainly of C-glycosyl apigenin derivatives, which are quite rare among Arecaceae species and may be used as phytochemical markers of *Oenocarpus*. Hydroxycinnamic acids were identified in both organs, as caffeoylquinic acids in leaflets, as caffeoylquinic and caffeoylshikimic acids in root extracts. CQA and flavones were the main drivers of antioxidant activity in the *Oenocarpus* roots and leaflets, respectively. The antioxidant activity of leaflet extracts was very promising, being at least as high as *E. oleracea* berries known as superfruit. These extracts could be valorized as a new non-cytotoxic source of antioxidants for dermatological or pharmaceutical applications.

## Figures and Tables

**Table 1:** Total Phenolic Content (TPC) of *O. bataua* and *O. bacaba* leaflet and root extracts shows total phenolic content (TPC) of *Oenocarpus* extracts according to extraction solvents and organs. Water extracts fromroots and leaflets had low TPC, ranging from 10 to 32 µg GAEq /mg DM (Table 1).

Extract	Organ	Palm	TPC (µg GAEq ^a^ /mg DM ^b^)	Statistical Significance ^c^ (*p* < 0.05)
**Water**	Leaflet	Obt ^d^	10.1 ± 0.8	E
		Obc ^e^	31.8 ± 5.4	C
	Root	Obt ^d^	9.6 ± 0.4	DE
		Obc ^e^	10.1 ± 0.8	E
**Acetone/Water (70/30 *v*/*v*)**	Leaflet	Obt ^d^	51.1 ± 1.7	AB
		Obc ^e^	63 ± 5.9	A
	Root	Obt ^d^	33.2 ± 2.4	BC
		Obc ^e^	28.4 ± 2.1	CD
**Methanol/Water (70/30 *v/v*)**	Leaflet	Obt ^d^	24.9 ± 1.6	CDE
		Obc ^e^	52 ± 6.5	AB
	Root	Obt ^d^	29.2 ± 2.1	C
		Obc ^e^	27.5 ± 1.2	CD

^a^ GAEq: Gallic acid equivalent; ^b^ DM: Dry Matter; ^c^ Extracts which share common letters are statistically identical using Fisher’s least significant difference test (*p* < 0.05); ^d^ Obt: *Oenocarpus bataua*; ^e^ Obc: *Oenocarpus bacaba*; *n* = 3 biological repetitions and error represent Standard Error of the Mean (SEM).

**Table 2:** Antioxidant activity of *Oenocarpus bataua* and *Oenocarpus bacaba* leaflet and root extracts.

Extract	Organ	Palm	DPPH ^a^		FRAP ^b^		ORAC_FL_^c^	
			(µmol TEq ^d^/g DM ^e^)	Statistical Significance ^f^ (*p* < 0.05)	(µmol Fe(II)Eq ^g^/g DM ^e^)	Statistical Significance ^f^ (*p* < 0.05)	(µmol TEq ^d^/g DM ^e^)	Statistical significance ^f^ (*p* < 0.05)
**Water**	Leaflet	Obt ^h^	68.9 ± 10.9	E	138 ± 28	F	229 ± 42	DE
		Obc ^i^	468.9 ± 218.5	ABC	480 ± 178	CDEF	610 ± 247	BCE
	Root	Obt ^h^	60.9 ± 18.7	E	128 ± 20	F	218 ± 28	E
		Obc ^i^	83.3 ± 10.8	DE	196 ± 8	EF	248 ± 17	CDE
**Acetone/Water (70/30 *v*/*v*)**	Leaflet	Obt ^h^	461 ± 35.8	AB	729 ± 23	ABC	1203 ± 168	A
		Obc ^i^	544.9 ± 106	A	1026 ± 218	A	1565 ± 183	A
	Root	Obt ^h^	288.7 ± 12.7	BCD	559 ± 44	BCD	720 ± 76	B
		Obc ^i^	252.3 ± 11.3	BCDE	512 ± 26	CDE	656 ± 66	B
**Methanol/Water (70/30 *v*/*v*)**	Leaflet	Obt ^h^	197.4 ± 39	CDE	327 ± 54	DEF	569 ± 38	BCDE
		Obc ^i^	389.7 ± 116.1	ABC	917 ± 231	AB	1268 ± 199	A
	Root	Obt ^h^	227.7 ± 33.9	CDE	418 ± 52	CDEF	627 ± 68	BC
		Obc ^i^	215.9 ± 12	CDE	441 ± 34	CDEF	579 ± 67	BCDE

^a^ DPPH: 2,2-diphenyl-1-picrylhydrazyl; ^b^ FRAP: ferric-reducing ability of plasma); ^c^ ORAC_FL_: oxygen radical absorbance capacity; ^d^ TEq: Trolox equivalent; ^e^ DM: Dry Matter; ^f^ Extracts which share common letters are statistically identical using Fisher’s least significant difference test (*p* < 0.05); ^g^ Eq: equivalent; ^h^ Obt: *Oenocarpus bataua*; ^i^ Obc: *Oenocarpus bacaba*. *n* = 3 biological repetitions and error represent Standard Error of the Mean (SEM).

**Table 3:** Cytotoxicity and cellular antioxidant activity of *O. bataua* and *O. bacaba* extracts in NHDF cells.

Extract	Organ	Palm	CAA Assay (Cellular Antioxidant Activity)			Cytotoxicity (MTT Assay) (µg/mL)
			EC_50_ (µg/mL)	µmoles Quercetin Eq ^a^/g DM ^b^	µmoles Quercetin Eq ^a^/100 g FW ^c^	
**Water**	Leaflet	Obt ^d^	65.7 ± 19.8	7.3 ± 2.2	365 ± 110	>400
		Obc ^e^	73.8 ± 11.3	6.5 ± 1.1	286 ± 47	Not toxic
	Root	Obt ^d^	71.9 ± 5.7	11.9 ± 1	252 ± 21	>400
		Obc ^e^	15.5 ± 1.3	15.5 ± 1.2	887 ± 69	Not toxic
**Acetone/Water (70/30 *v*/*v*)**	Leaflet	Obt ^d^	13.8 ± 2.3	100.9 ± 17	5028 ± 847	Not toxic
		Obc ^e^	24.1 ± 4.9	49.7 ± 11.5	2172 ± 502	>200
	Root	Obt ^d^	21.7 ± 3.2	34.7 ± 4.8	734 ± 101	>100
		Obc ^e^	10.4 ± 0.6	29.3 ± 1.9	1670 ± 106	Not toxic
**Methanol/Water (70/30 *v*/*v*)**	Leaflet	Obt ^d^	44.8 ± 0.3	19.7 ± 0.2	984 ± 7	Not toxic
		Obc ^e^	30 ± 3	24.2 ± 2.4	1057 ± 104	Not toxic
	Root	Obt ^d^	29.3 ± 2.9	35.3 ± 3.8	751 ± 80	Not toxic
		Obc ^e^	12.9 ± 1	31.5 ± 2.5	1799 ± 145	Not toxic

^a^ Eq: equivalent; ^b^ DM: Dry Matter; ^c^ FW: fresh weight; ^d^ Obt: *Oenocarpus bataua*; ^e^ Obc: *Oenocarpus bacaba*. *n* = 3 repetitions and error represent Standard Deviation (SD).

**Table 4:** Identification of main components of root and leaflet extracts of *Oenocarpus bacaba* and *Oenocarpus bataua.*

No.	*t*_R_ (min)	UV λ_max_ (nm)	Negative Mode MS	Negative Mode MS^2^	Positive Mode MS	Positive Mode MS^2^	Tentative Identity	Abbreviation	Extracts
1	4.2	238, 295sh, 321	353 [M − H]^−^	191, 179	377 [M + Na]^+^	377, 353, 163, 145	3-Caffeoylquinic acid	3-CQA	Roots, leaflets
2	7.9	237, 286sh, 322	353 [M − H] ^−^	173	377 [M + Na]^+^	377, 353, 163, 145	4-Caffeoylquinic acid	4-CQA	Roots, leaflets
3	9.1	239, 295sh, 323	353 [M − H] ^−^	191	377 [M + Na]^+^	377, 353, 163, 145	5-Caffeoylquinic acid	5-CQA	Roots, leaflets
4	12.2	239, 295sh, 325	335 [M − H] ^−^	291, 179, 161, 135	359 [M + Na]^+^	359, 163, 145	4-Caffeoylshikimic acid	4-CSA	Roots
5	14.2	239, 295sh, 325	335 [M − H] ^−^	317, 291, 179	359 [M + Na]^+^	359, 163, 145	5-Caffeoylshikimic acid	5-CSA	Roots
6	16.2	239, 295sh, 325	335 [M − H] ^−^	317, 291, 179, 161, 135	359 [M + Na]^+^	359, 163, 145	Caffeoylshikimic acid	CSA	Roots
7	16.1	239, 270, 335	593 [M − H] ^−^	503, 473, 383, 353	595 [M + H]^+^	595, 577, 457, 427, 317	6,8-di-C-hexosyl apigenin	Di-Glc-Api	Leaflets
8	16.8	239, 270, 335	673 [M − H] ^−^	593, 575, 503, 473, 413, 383, 353	713 [M + K]^+^	633, 593, 543, 513, 423, 393, 351	6,8-di-C-hexosyl apigenin sulfate	Di-Glc-Api-Sulf	Leaflets
9	17.8	239, 270, 335	673 [M − H] ^−^	593, 503, 473, 383, 353	713 [M + K]^+^	633, 593, 543, 513, 483, 423, 393, 363, 351	6,8-di-C-hexosyl apigenin sulfate	Di-Glc-Api-Sulf	Leaflets
10	21.2	238, 272, 339	563 [M − H] ^−^	473, 443, 383, 353	–	–	6-C-hexosyl-8-C-pentosyl apigenin isomer		Leaflets
11	22.1	240, 270, 342	447 [M − H] ^−^	447, 429, 411, 357, 327, 299	449 [M + H]^+^	449, 413, 383	8-C-glucosyl luteolin (orientin) *		Leaflets
12	22.3	238, 272, 339	563 [M − H] ^−^	503, 473, 443, 413, 383, 353	–	–	6-C-pentosyl-8-C-hexosyl apigenin isomer		Leaflets
13	22.9	241, 270, 346	447 [M − H] ^−^	447, 429, 411, 357, 327, 299	449 [M + H]^+^	431, 413, 395, 383, 353, 329, 299	6-C-glucosyl luteolin (isoorientin) *		Leaflets
14	23.2	238, 272, 340	563 [M − H] ^−^	473, 443, 383, 353	–	–	6-C-hexosyl-8-C-pentosyl apigenin isomer		Leaflets
15	24.9	241, 271, 336	563 [M − H] ^−^	443, 383, 353, 323	–	–	6-C-hexosyl-8-C-pentosyl apigenin isomer		Leaflets
16	25.6	238, 277, 335	563 [M − H] ^−^	503, 473, 383, 353	–	–	6-C-pentosyl-8-C-hexosyl apigenin isomer		Leaflets
17	26.4	241, 270, 337	431[M − H] ^−^	431, 413, 395, 341, 311, 283	433 [M + H]^+^	397, 379, 367, 337, 313, 295, 283	6-C-glucosyl apigenin (isovitexin) *		Leaflets

* Structure confirmed using synthetic compounds.

## Supplementary Materials

Supplementary materials can be found at http://www.mdpi.com/1422-0067/17/7/1014/s1.

## Abbreviations

Obt	*Oenocarpus bataua*
Obc	*Oenocarpus bacaba*
Eo	*Euterpe oleracea*
DM	Dry matter
FW	Fresh weight
HCA	Hydroxycinnamic acid
CQA	Caffeoylquinic acid
CSA	Caffeoylshikimic acid
NHDF	Normal human dermal fibroblasts
ORAC	Oxygen radical absorbance capacity
FRAP	Ferric-reducing ability of plasma
DPPH	2,2-Diphenylpicrylhydrazyl
TPC	Total phenolic content
CAA	Cellular antioxidant activity
W	Water
A	Acetone/water, 70/30, *v*/*v*
M	Methanol/water, 70/30, *v*/*v*
GAEq	Gallic acid equivalent
TEq	Trolox equivalent
Eq	Equivalent
LC-MSn	Liquid chromatography coupled with mass spectrometry
PCA	Principal Component Analysis
