# Supplementary material for: Oenocarpus bacaba and Oenocarpus bataua Leaflets and Roots: A New Source of Antioxidant Compounds
Source: Int J Mol Sci. 2016 Jun 27;17(7):1014. doi: 10.3390/ijms17071014 (PMC4964390; doi:10.3390/ijms17071014)
Supplement: Supplementary file 1 [file ijms-17-01014-s001.pdf]

Supplementary Materials: *Oenocarpus bacaba* and *Oenocarpus bataua* Leaflets and Roots: A New Source of Antioxidant Compounds

Louis-Jérôme Leba, Christel Brunschwig, Mona Saout, Karine Martial, Didier Bereau and Jean-Charles Robinson

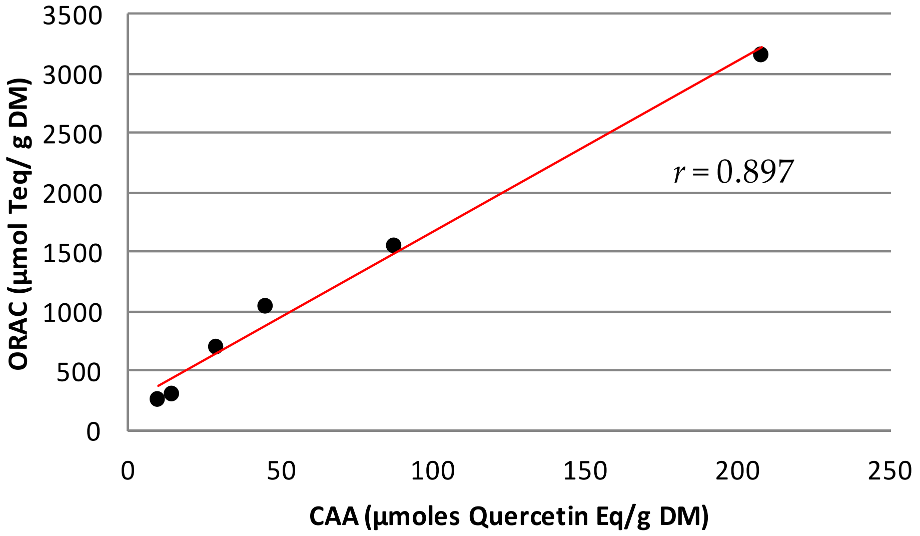

Figure S1. Correlation between ORAC and CAA activity. Correlations have been calculated using a Pearson's *r* correlation coefficient ($p < 0.05$). n: 3 biological repetitions by palm species.

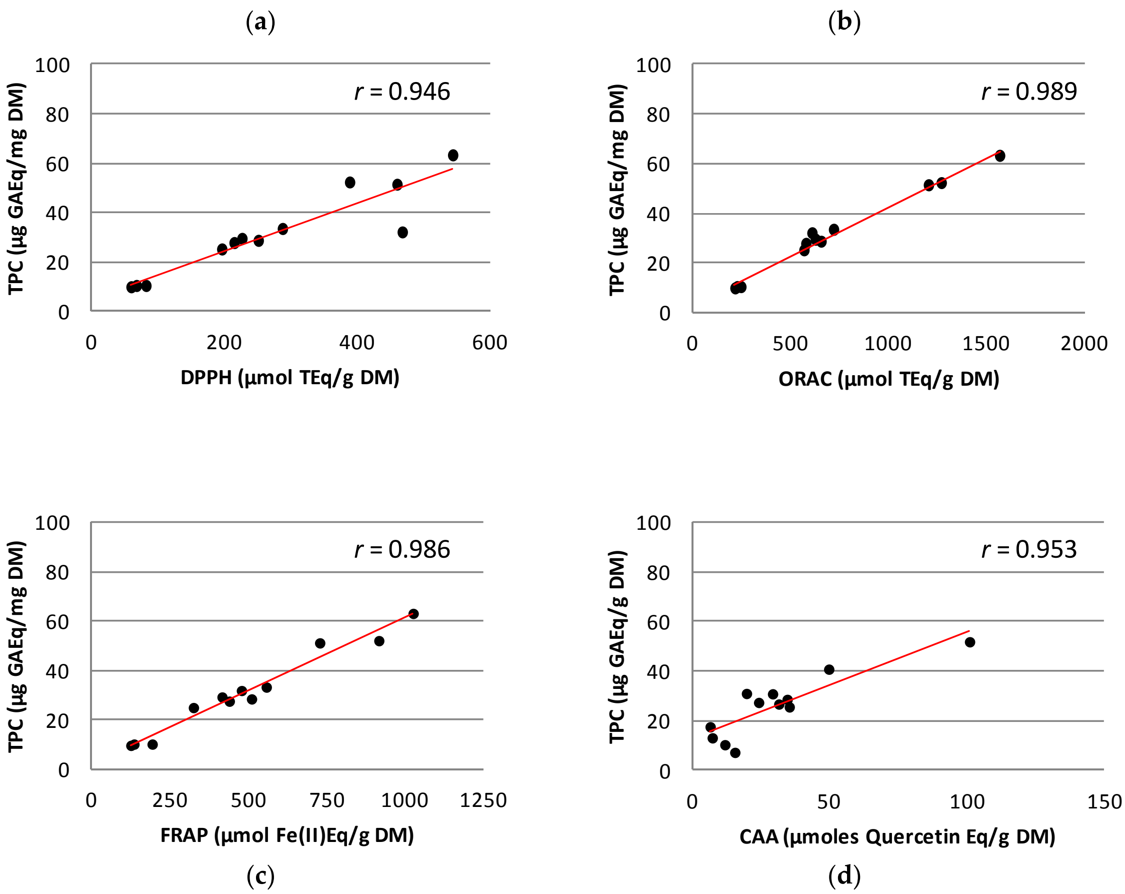

Figure S2. Correlations between Total Phenolic Content and, (**a**) DPPH, (**b**) FRAP, (**c**) ORAC (**d**) CAA of *O. bataua* and *O. bacaba* root and leaflet extracts. Correlations have been calculated using a Pearson's *r* correlation coefficient ($p < 0.05$). n: 3 biological repetitions by palm species.

Table S1. Palm extracts classification.

Classification	TPC			DPPH			FRAP			ORAC		
	**Organ**	**Extract**	**Content** [a]	**Organ**	**Extract**	**Activity** [b]	**Organ**	**Extract**	**Activity** [c]	**Organ**	**Extract**	**Activity** [d]
**Poor extracts**	R	Obt-W	9,6	R	Obt-W	60,9	R	Obt-W	128	R	Obt-W	218
	L	Obt-W	10,1	L	Obt-W	68,9	L	Obt-W	138	L	Obt-W	229
	R	Obc-W	10,1	R	Obc-W	83,3	R	Obc-W	196	R	Obc-W	248
**Intermediate extracts**	L	Obt-M	24,9	L	Obt-M	197,4	L	Obt-M	327	L	Obt-M	569
	R	Obc-M	27,5	R	Obc-M	215,9	R	Obt-M	418	R	Obc-M	579
	R	Obc-A	28,4	R	Obt-M	227,7	R	Obc-M	441	L	Obc-W	610
	R	Obt-M	29,2	R	Obc-A	252,3	L	Obc-W	480	R	Obt-M	627
	L	Obc-W	31,8	R	Obt-A	288,7	R	Obc-A	512	R	Obc-A	656
	R	Obt-A	33,2	L	Obc-M	389,7	R	Obt-A	559	R	Obt-A	720
**Good extracts**	L	Obt-A	51,1	L	Obt-A	461	L	Obt-A	729	L	Obt-A	1203
	L	Obc-M	52	L	Obc-W	468,9	L	Obc-M	917	L	Obc-M	1268
	L	Obc-A	63	L	Obc-A	544,9	L	Obc-A	1026	L	Obc-A	1565

Obt: *Oenocarpus bataua*; Obc: *Oenocarpus bacaba*; A: Acetone/water; M: Methanol/water; W: Water, R: Root; L: Leaflet; [a] Content in µg GAEq/mg DM; [b] Activity in µmol TEq/g DM; [c] Activity in µmol TEq/g DM; [d] µmol TEq/g DM. Classification have been performed using the reproducible hierarchical positioning of the extract in the 4 assays and their activity value.

Table S2. Total phenolic content and chemical activities (DPPH, FRAP, ORAC) of *Oenocarpus bataua, Euterpe oleracea* berries and green tea leaves.

**Extract**	**Organ**	**Assay**	**TPC**	**DPPH**	**FRAP**	**ORAC**
		**Palm**	**(µg GAEq/mg DM)**	**(µmol TEq/g DM)**	**(µmol Fe(II)Eq/g DM)**	**(µmol TEq/g DM)**
**Water**	berry	Obt (1)	8.2 ± 0.5	32 ± 0.2	137 ± 7.6	166 ± 12.4
	leaf	Green tea (1)	99.5 ± 8.6	748 ± 51.9	1911 ± 81.6	1348 ± 45.4
**Acetone/Water**	berry	Obt (1)	32.7 ± 5.8	235 ± 41.9	480 ± 85.5	379 ± 67.5
		Obt (2)	32.2 ± 0.8	241 ± 12.9	706 ± 11	170 ± 1.4
		Eo (2)	38.7 ± 4.6	257 ± 22.5	503 ± 5.8	453 ± 4.5
	leaf	Green tea (1)	126.2 ± 2	1185 ± 33.9	2686 ± 106.8	2167 ± 19
**Methanol/Water**	berry	Obt (1)	30 ± 2.9	201 ± 19.6	469 ± 45.9	362 ± 35.5
	leaf	Green tea (1)	98.3 ± 8	1045 ± 68.7	26775 ± 142.4	2366 ± 30.6

Eo: *Euterpe oleracea*; Obt: *Oenocarpus bataua*; DM: Dry Mater; GAEq: Gallic acid equivalent; TEq: Trolox equivalent; Eq: equivalent. (1) data from Leba et al. (2014); (2) data from Rézaire et al. (2014). n = 3 repetitions and error represent Standard Error of the Mean (SEM)
